# An evaluation of the competencies of primary health care clinic nursing managers in two South African provinces

**DOI:** 10.3402/gha.v9.32486

**Published:** 2016-12-09

**Authors:** Pascalia O. Munyewende, Jonathan Levin, Laetitia C. Rispel

**Affiliations:** 1 Centre for Health Policy & Medical Research Council Health Policy Research Group, School of Public Health, Faculty of Health Sciences, University of the Witwatersrand, Johannesburg, South Africa; 2 Division of Epidemiology and Biostatistics, School of Public Health, Faculty of Health Sciences, University of the Witwatersrand, Johannesburg, South Africa; 3 DST/NRF South African Research Chair, School of Public Health, Faculty of Health Sciences, University of the Witwatersrand, Johannesburg, South Africa

**Keywords:** competencies, nursing management, primary health care clinics, training, South Africa

## Abstract

**Background:**

Managerial competencies to enhance individual and organisational performance have gained currency in global efforts to strengthen health systems. Competent managers are essential in the implementation of primary health care (PHC) reforms that aim to achieve universal health coverage.

**Objective:**

To evaluate the competencies of PHC clinic nursing managers in two South African provinces.

**Design:**

A cross-sectional study was conducted in two South African provinces. Using stratified random sampling, 111 PHC clinic nursing managers were selected. All supervisors (*n*=104) and subordinate nurses (*n*=383) were invited to participate in the survey on the day of data collection. Following informed consent, the nursing managers, their supervisors, and subordinate nurses completed a 40-item, 360-degree competency assessment questionnaire, with six domains: communication, leadership and management, staff management, financial management, planning and priority setting, and problem-solving. Standard deviations, medians, and inter-quartile ranges (IQRs) were computed separately for PHC nursing managers, supervisors, and subordinate nurses for competencies in the six domains. The Tinsley and Weiss index was used to assess agreement between each of the three possible pairs of raters.

**Results:**

A 95.4% response rate was obtained, with 105 nursing managers in Gauteng and Free State completing the questionnaires. There was a lack of agreement about nursing managers’ competencies among the three groups of raters. Overall, clinic nursing managers rated themselves high on the five domains of communication (8.6), leadership and management (8.67), staff management (8.75), planning and priority setting (8.6), and problem-solving (8.83). The exception was financial management with a median score of 7.94 (IQR 6.33–9.11). Compared to the PHC clinic managers, the supervisors and subordinate nurses gave PHC nursing managers lower ratings on all six competency domains, with the lowest rating for financial management (supervisor median rating 6.56; subordinate median rating 7.31).

**Conclusion:**

The financial management competencies of PHC clinic nursing managers need to be prioritised in continuing professional development programmes.

## Introduction

Globally, managerial competencies to enhance individual and organisational performance have gained currency in efforts to strengthen national health systems and improve population health outcomes (1–3). The 2007 World Health Organization (WHO) conceptual framework for strengthening leadership and management capacity includes appropriate managerial competencies needed for effective health service delivery (1). ‘Competency’ is defined as the technical skills, knowledge, and attitudes required to perform a job (2). Management competencies in strategic, financial, and human resource management; service delivery innovation; client orientation and customer focus; and communication are essential for achieving organisational goals (4). In complex health systems, health care managers require additional competencies to complement the generic management functions of planning, directing, coordinating, and controlling to be effective (5). This is because managerial competencies are important for optimal patient care and continuous quality of health improvement (3, 6), while competent managers play a key role in the implementation of universal health coverage reforms (7, 8). The International Council of Nurses and the World Health Professions Alliance advocate for competence-based education for health service managers that take account of career stage, roles and responsibilities, work context, and setting (9, 10), through in-service training, short courses, diplomas, and degree training programmes (2, 11).

However, in many low- and middle-income countries (LMICs), the health workforce crisis and suboptimal functioning of health systems are compounded by the lack of competent managers (12–14). Managerial incompetence has negative consequences for service delivery, well-being, and retention of health workers and health system performance (13, 15, 16).

A significant body of literature exists on the description, identification, classification, and measurement of the most appropriate competencies for managers, including nursing managers (13, 15, 17–20). Several authors have suggested that nursing management competencies should include communication, team building, planning, priority setting and problem-solving, performance, health programme, and resource management (2, 6, 19–21), especially financial management (22, 23). Change management and community collaboration are other essential competencies required at the district level (18).

Measuring competencies in a reliable and valid way is complex (4). Approaches include personality and psychometric tests (17, 18, 24), self-ratings (25), formal examinations (26), case studies (27), and vignettes of management challenges (28). In South Africa, personality and psychometric tests using different types of Likert scales are used to measure competencies of executive managers (17, 18).

Some studies have used the 360-degree evaluation approach to determine the mastering of core competencies for medical doctors (29) and nursing managers (30). The 360-degree approach involves self-assessment by individuals on key performance areas and assessments by their supervisors and direct reports or peers (31). The success of the 360-degree method depends on the raters (31). These assessments aim to increase the individuals’ awareness of their own competencies and how those competencies are viewed by others. Respondents are encouraged to list their weak competency areas for further training (25). Although this practice is common in businesses (32), the method is increasingly being used in public health, with health care managers being evaluated by their supervisors, subordinates, or even patients (26, 33, 34).

Ongoing assessment of health managers’ competencies is associated with health systems strengthening, staff retention, and job satisfaction (7). Competency assessments enable managers to enhance the skills needed to perform in complex health care systems (19). However, studies on managerial competencies tend to focus on district or hospital managers, and there is a dearth of studies focusing on the competencies of primary health care (PHC) clinic nursing managers (7). A small South African qualitative study on nursing managers’ competencies, which cannot be generalised, concluded that the success of PHC re-engineering relies on skills and knowledge in health service planning, organisation, change management and leadership, conducting community health needs assessments, and basic statistics to interpret health information (17). Furthermore, the study did not include the views of the PHC managers (17).

The importance of PHC and its managers was re-emphasised in the 2015 South African White Paper on the national health insurance (NHI) system (35). The proposed NHI reforms are based on PHC, and nursing managers will play a key role in its implementation as they operate at the interface between care processes and overall health system goals (35).

The evaluation of the competencies of nursing managers is important for determining their training needs and to benchmark the performance of PHC facilities (18). Competent managers facilitate the implementation of health care reforms, through ensuring staff participation and managing complex change. In light of this, and the dearth of empirical information on the managerial competencies of PHC nursing managers, the aim of this study was to evaluate the competencies of clinic nursing managers in two South African provinces by using a 360-degree assessment method that included their supervisors and those nurses reporting to them (subordinate nurses).

## Methods

### Study sites and setting

In 2012, a cross-sectional study was conducted in two South African provinces (15, 36) – Gauteng (an urban province) and Free State (a mixed urban–rural province) – selected on the basis of geographical access to the researchers, existing health authority approval, and financial considerations (15, 36). In 2012, there were 343 PHC clinics in Gauteng province (37) and 256 PHC clinics in Free State province (38).

### Study population

The study population consisted of professional nurses, with a 4-year qualification in nursing, registered with the South African Nursing Council, and in charge of an 8 hour (day) PHC clinic. The PHC nursing managers are responsible for clinic management, including health care service delivery, staff, and implementation of policies (15, 36). Non-nursing managers in charge of clinics operating for more than 8 hour a day, weekend or mobile clinics and community health centres, and those linked to hospitals were excluded from the study.

### Sampling

For the study sampling approach, we drew up a list of 8 hour PHC clinics in each of the health districts: six in Gauteng and five in Free State province. Once this list was obtained, stratified random sampling proportional to the number of clinics in each district was used to calculate the number of clinics to be included in the study sample – 62 and 49 clinics were sampled from Gauteng and Free State provinces, respectively. In each sampled clinic, a self-administered 360-degree competency evaluation questionnaire was given to the PHC nursing managers, their supervisors (*n*=104), and subordinate nurses (*n*=383). The number of subordinates ranged from 1 to 7, with a median of 4.

### Development of the 360-degree competency evaluation instrument

Following a review of the literature (7, 18), a 360-degree competency evaluation instrument consisting of 40 items was developed. The 360-degree assessment was selected to increase the individual clinic managers’ awareness of their own competencies, and how those competencies are viewed by others – in this case, their immediate supervisors and their nursing colleagues who work with them and report to them.

The tool comprises six subscales relating to daily clinic management tasks. They are as follows: *communication* (five items) examines whether the clinic manager listens attentively to the concerns of others, can write reports, and is able to share ideas with staff and other stakeholders. *Leadership and management* (six items) assesses whether the clinic manager is visionary, implements the National Department of Health's 10-point plan, and knows when to consult the relevant people on the strategic objectives of the clinic. *Staff management* (eight items) examines aspects of people management (e.g. absenteeism) and in-service training. *Financial management* (nine items) examines the clinic manager's ability to manage the budget in line with the financial legislation, whether the asset register is up to date, and develops realistic budget projections. *Planning and priority setting* (six items) contains dimensions of information management, prioritisation of tasks, and identification of community health needs. Lastly, the *problem-solving* subscale (six items) questions whether the clinic manager monitors the work environment for risks that could impact staff and patients, manages clinic emergencies, and implements corrective action for potential risks and problems. Each of the items was measured on a 10-point scale (where 1 being low skills, i.e. more training is needed, and 10 being very high skills, i.e. no training is needed). The information sheet and initial briefing to the PHC clinic managers emphasised that the rating was linked to their perceptions of competencies and training needs (Table 1).

**Table 1:** 360 degree evaluation of the job-related skill perceptions of PHC nursing managers

	My skills										
		
Communication	Low skills						Very high skills				Don't know
1. I listen attentively to the concerns of others	1	2	3	4	5	6	7	8	9	10	DN
2. I can share my ideas about improving services with the clinic supervisor	1	2	3	4	5	6	7	8	9	10	DN
3. I am able to write reports to meet the needs of different audiences	1	2	3	4	5	6	7	8	9	10	DN
4. I am able to communicate the specific programme targets to my staff	1	2	3	4	5	6	7	8	9	10	DN
5. I am able to inform community representatives about all the relevant clinic issues	1	2	3	4	5	6	7	8	9	10	DN
Leadership and management	Low skills						Very high skills				Don't know
6. I can implement my vision for health improvements in this clinic	1	2	3	4	5	6	7	8	9	10	DN
7. I am able to implement the 10-point plan of the NDOH for 2009–2014	1	2	3	4	5	6	7	8	9	10	DN
8. I know when to consult clinic employees when making strategic decisions	1	2	3	4	5	6	7	8	9	10	DN
9. I can deal with difficult patients and their relatives	1	2	3	4	5	6	7	8	9	10	DN
10. I encourage my staff to display a caring attitude to patients according to the Batho Pele principles	1	2	3	4	5	6	7	8	9	10	DN
11. I ensure that the risk of infection to patients in this clinic is minimised	1	2	3	4	5	6	7	8	9	10	DN
Staff management	Low skills						Very high skills				Don't know
12. I am able to measure clinical care programmes against National Core Standards	1	2	3	4	5	6	7	8	9	10	DN
13. I ensure that my staff receive ongoing in-service training to perform their nursing tasks	1	2	3	4	5	6	7	8	9	10	DN
14. I ensure that staff are knowledgeable about what is expected from them at work	1	2	3	4	5	6	7	8	9	10	DN
15. I supervise my staff through effective performance management for the achievement of health system goals	1	2	3	4	5	6	7	8	9	10	DN
16. I am able to delegate work to staff in an emergency context	1	2	3	4	5	6	7	8	9	10	DN
17. I have a system in place to manage staff absenteeism	1	2	3	4	5	6	7	8	9	10	DN
18. I provide constructive feedback to staff	1	2	3	4	5	6	7	8	9	10	DN
19. I monitor individual's performance in response to allocated work	1	2	3	4	5	6	7	8	9	10	DN
Financial management	Low skills						Very high skills				Don't know
20. I manage the clinic budget in line with the relevant financial legislation	1	2	3	4	5	6	7	8	9	10	DN
21. I make sure that the asset register in my clinic is up to date	1	2	3	4	5	6	7	8	9	10	DN
22. I take prompt action when equipment is out of order	1	2	3	4	5	6	7	8	9	10	DN
23. I take prompt action when contractors fail to deliver against their service-level agreement	1	2	3	4	5	6	7	8	9	10	DN
24. I engage staff about how best to use financial resources	1	2	3	4	5	6	7	8	9	10	DN
25. I educate staff about financial issues that impact the clinic	1	2	3	4	5	6	7	8	9	10	DN
26. I can develop realistic budget projections based on previous expenditure	1	2	3	4	5	6	7	8	9	10	DN
27. I ensure that all the necessary expenses are included in the budget	1	2	3	4	5	6	7	8	9	10	DN
28. I am able to evaluate programme performance in relation to expenditure	1	2	3	4	5	6	7	8	9	10	DN
Planning and priority setting	Low skills						Very high skills				Don't know
29. I use clinic statistics to motivate for financial and human resources	1	2	3	4	5	6	7	8	9	10	DN
30. I involve stakeholders in determining which needs are the most important to improve clinic performance	1	2	3	4	5	6	7	8	9	10	DN
31. I generally defend the interests of the clinic	1	2	3	4	5	6	7	8	9	10	DN
32. I always carry out the most urgent tasks first	1	2	3	4	5	6	7	8	9	10	DN
33. I follow through on agreed tasks and priorities	1	2	3	4	5	6	7	8	9	10	DN
34. I am able to identify community health needs in my catchment area for use in strategic planning	1	2	3	4	5	6	7	8	9	10	DN
Problem-solving	Low skills						Very high skills				Don't know
35. I monitor the work environment for potential safety issues that could impact staff and patients	1	2	3	4	5	6	7	8	9	10	DN
36. I remain calm and composed in emergency situations	1	2	3	4	5	6	7	8	9	10	DN
37. I implement corrective action in a timely manner in cases of non-compliance	1	2	3	4	5	6	7	8	9	10	DN
38. I can resolve conflicts among staff	1	2	3	4	5	6	7	8	9	10	DN
39. I can identify root causes of problems in the clinic	1	2	3	4	5	6	7	8	9	10	DN
40. I gather sufficient information prior to solving a problem	1	2	3	4	5	6	7	8	9	10	DN

Supervisors and subordinate nurses rated their perceptions of the clinic managers’ competencies separately. The phrasing of the questions was adjusted for supervisors and subordinate nurses and made it clear that they were rating the competencies of the PHC clinic managers, and their training needs (where relevant).

After the development of the study instrument, a 22-member expert group, consisting of academics, government and private sector health service managers, national nursing association representatives, health systems management and policy experts, discussed and reviewed the content of the questionnaire at a workshop organised for this purpose. Hence, this expert group ensured both content and face validity of the questionnaire.

The expert panel recommended improvements to the questionnaire, and a consensus was reached on the proposed changes. Prior to fieldwork, the instrument was pilot-tested with three PHC nursing managers and their clinic supervisors and subordinate nurses to determine clarity of the questions and the time needed to complete the questionnaire (39). No further modifications were necessary. The nine pilot respondents were excluded from the main study.

### Data collection

Clinic nursing managers were contacted in July 2012 to encourage their involvement and to schedule the date for data collection. Information meetings were conducted with the PHC nursing managers to describe the study components. Following informed consent, the clinic nursing managers were given the self-administered 360-degree competency evaluation questionnaire to complete. Subordinate nurses were also approached and requested to evaluate the competencies of their clinic managers. Clinic supervisors were approached at their offices and were asked to rate the clinic managers who report to them. The questionnaire took around 10 min to complete.

A research assistant collected the questionnaires on the day of completion. The researcher conducted quality checks to confirm the completeness of the questionnaires. Steps were taken to ensure that each clinic manager rated herself and further was rated by a clinic supervisor and her subordinate nurses. Double data entry from the questionnaires was done by a data capturing company. Data were cleaned and checked for inconsistencies before being imported into STATA^®^13 for analysis. A ‘don't know’ response was set as a ‘missing’ value in STATA.

### Data management and analysis

Cronbach's alpha coefficients were calculated separately for each of the three groups of respondents – nursing managers, supervisors, and subordinate nurses for each of the six domains – in order to confirm the reliability of the scales. As a further confirmation, for each of the three groups of respondents and for each of the six domains, an exploratory factor analysis was carried out to confirm that the items in the domain were summarised by a single construct. In addition, an exploratory factor analysis was carried out on the combined responses from all the domains to check whether the data suggested six separate domains.

The mean score was calculated for each domain and for each respondent group. The responses on each domain were summarised using means, standard deviations, medians, and inter-quartile ranges (IQRs), separately for nursing managers, supervisors, and subordinate nurses. To determine the agreement between each of the three possible pairs of type of respondents, we used the Tinsley and Weiss index (40). This index measures the consistency with which inter-raters reach agreement about a subject or ‘the extent to which the different judges tend to assign the same rating to each object’ (40, p. 98). In calculating the index, we assumed that any ratings that differed by at most 1 were in agreement, while any ratings that differed by more than 1 were not in agreement. We calculated agreement between two raters and the hypothetical probability of chance agreement, and then we estimated the Bland–Altman limits of agreement which provide a quantitative measurement of how closely two groups of raters agree on certain facts (41).

We defined a ‘PHC nursing manager’ as competent and not requiring training if the average score was at least 8 or above out of 10 on all the competency domains. This value was selected as the majority of scoring systems in educational settings use 80% for distinctions, and this value is used as a proxy for competence. To determine the need for additional training, we looked at the proportion of managers who rated themselves and were also rated less than 8/10 by their supervisors and subordinates.

### Ethical considerations

The study is part of a larger doctoral research project to examine the nature and dynamics of nursing management at PHC level. The University of the Witwatersrand's Human Research Ethics Committee (M 12 02 48) granted ethical approval. Permission for the study was obtained from the relevant health care authorities in the two provinces. Prior to fieldwork, an information sheet was given to each study participant, and informed consent was obtained. Each participant was informed of the study's voluntary nature and that confidentiality and anonymity would be maintained.

## Results

A 95.4% (*n*=105) response rate was achieved from PHC nursing managers (Gauteng, *n*=57 and Free State, *n*=48) who completed the 360-degree competency evaluation. The average age of PHC clinic nursing managers was 49 years (SD: 7.8). With regard to gender, the majority of study participants in both provinces were female (92%), married (63%), and were employed in a permanent position (89%) (15). Of the PHC nursing managers, 36% had about 21–30 years of professional nursing experience and 76% had formal qualifications in PHC clinic management (15).

### Nursing managers’, supervisors’, and other nurses’ scores on competency categories

Table 2 highlights Cronbach's alpha coefficients of greater than 0.8 for all domains (communication, leadership and management, staff management, financial management, planning and priority setting, and problem-solving) for all three groups of respondents (clinic nursing managers, supervisors, and subordinate nurses), confirming the reliability of the scales. Furthermore, exploratory factors analyses confirmed that for each group and dimension, the items were well explained by a single construct with similar positive loadings for all items. The exception was financial management which required two factors for nursing managers. The fact that all loadings were large and positive for the first factor and that financial management was explained by a single factor for supervisors and nurses was acceptable. An overall factor analysis on all items suggested that, in fact, five factors might be sufficient (rather than the six domains). The factor loadings for the five-factor model did not show an easily interpretable pattern, not even after carrying out a factor rotation.

**Table 2:** Summary of scales by type of respondents

Scale	Statistic	Nursing managers	Supervisors	Other nurses reporting to nurse manager
A: Communication (five items)	N	105	104	109
	Mean (SD)	8.51 (1.25)	7.33 (1.75)	7.48 (1.93)
	Median (IQR)	8.6 (7.8–9.6)	7.6 (6.3–8.6)	7.9 (6.2–9.0)
	Cronbach's α	0.84	0.92	0.96
B: Leadership and management (six items)	N	105	104	109
	Mean (SD)	8.55 (1.14)	7.32 (1.77)	7.68 (1.69)
	Median (IQR)	8.67 (7.83–9.67)	7.83 (6.17–8.58)	8.0 (6.58–9.0)
	Cronbach's α	0.86	0.93	0.96
C: Staff management (eight items)	N	105	104	109
	Mean (SD)	8.54 (1.26)	7.22 (1.89)	7.56 (1.73)
	Median (IQR)	8.75 (7.88–9.62)	7.69 (6.12–8.56)	7.81 (6.61–8.75)
	Cronbach's α	0.90	0.95	0.97
D: Financial management (nine items)	N	102	104	109
	Mean (SD)	7.68 (1.77)	6.64 (1.94)	7.25 (1.93)
	Median (IQR)	7.94 (6.33–9.11)	6.56 (5.22–8.11)	7.31 (6.09–8.72)
	Cronbach's α	0.90	0.95	0.96
E: Planning and priority setting (six items)	N	103	104	109
	Mean (SD)	8.41 (1.38)	7.26 (1.75)	7.54 (1.83)
	Median (IQR)	8.6 (7.33–9.83)	7.58 (6.08–8.67)	7.76 (6.57–9.08)
	Cronbach's α	0.87	0.91	0.95
F: Problem-solving (six items)	N	105	104	109
	Mean (SD)	8.68 (1.22)	7.36 (1.80)	7.36 (1.91)
	Median (IQR)	8.83 (7.67–9.83)	7.67 (6.17–8.75)	7.75 (6.27–8.89)
	Cronbach's α	0.93	0.94	0.96

In general, clinic nursing managers rated themselves high on all domains except for financial management. The majority of nursing managers rated themselves as competent on the other five domains, while the median on financial management was 7.94 (IQR 6.33–9.11).

The majority of supervisors gave lower ratings on all the competency domains, compared to the clinic managers or their subordinates (mean 6.64; SD: 1.94) for the financial management domain. The subordinates also rated their clinic managers lower in all domains, compared to the self-ratings by these managers.

### Agreement between each possible pair of raters

Table 3 lists the six competency domains and the agreement on each domain between each of the three possible pairs of raters (i.e. manager and supervisor, manager and subordinates, and supervisor and subordinates). In all cases, there was less than 50% agreement. The strength of agreement is measured by the Tinsley–Weiss T-index, where perfect agreement would lead to a T-index of 1. The largest value of T-index is 0.25, which shows that the level of agreement between any of the three pairs is very poor. In addition, the Bland–Altman limits of agreement are very wide; for example, for communication, the limits of agreement for subordinates and supervisors are −4.44 to 4.72, which shows that the rating of a nurse compared to that of a supervisor could be anywhere between 4.44 units lower to 4.72 units higher (on a scale of 1 to 10). The mean difference between the ratings of the two members of each pair shows a systematic difference in each case – therefore, in the same example, subordinates rated nursing managers 0.14 units higher than supervisors did. In each domain, the rating of the nurse manager is at least 1 unit higher than that of the supervisor.

**Table 3:** Agreement between each possible pair of raters

Scale	Statistic	Nurse manager versus supervisor (*n*=98)	Nurse manager versus other nurses (*n*=103)	Other nurses versus supervisor (*n*=103)
A: Communication (five items)	Agreements Na	32	40	37
	T-index	0.11	0.22	0.18
	Mean Diff (SD)	1.28 (2.01)	1.08 (2.19)	0.14 (2.29)
	Limits of agreement	−2.74; 5.30	−3.30; 5.46	−4.44; 4.72
B: Leadership and management (six items)	Agreements Na	42	37	38
	T-index	0.25	0.19	0.19
	Mean Diff (SD)	1.26 (2.02)	0.89 (1.94)	0.34 (2.12)
	Limits of agreement	−2.78; 5.30	−2.99; 4.77	−3.90; 4.58
C: Staff management (eight items)	Agreements Na	40	36	37
	T-index	0.22	0.17	0.18
	Mean Diff (SD)	1.33 (2.10)	1.01 (1.99)	0.31 (2.17)
	Limits of agreement	−2.87; 5.53	−2.97; 4.99	−4.03; 4.65
D: Financial management (nine items)	Agreements Na	32	34	29
	T-index	0.11	0.15	0.08
	Mean Diff (SD)	1.11 (2.47)	0.41 (2.51)	0.58 (2.51)
	Limits of agreement	−3.83; 6.05	−4.61; 5.43	−4.44; 5.60
E: Planning and priority setting (six items)	Agreements Na	30	40	35
	T-index	0.086	0.22	0.15
	Mean Diff (SD)	1.13 (2.14)	0.88 (2.11)	0.28 (2.23)
	Limits of agreement	−3.15; 5.41	−3.34; 5.10	−4.18; 4.74
F: Problem-solving (six items)	Agreements Na	36	32	31
	T-index	0.17	0.12	0.10
	Mean Diff (SD)	1.31 (2.04)	1.36 (2.11)	−0.001 (2.31)
	Limits of agreement	−2.77; 5.39	−2.86; 5.58	−4.62; 4.62

*Note*. Supervisor and other nurses’ ratings were omitted if the nursing managers had not rated themselves.

### Proportion of nursing managers rated below 8/10

Table 4 highlights the proportion of nursing managers rated below 8/10 on all the competency dimensions. Overall, the proportion of supervisors who rated nursing managers less than 8/10 was greater than that of the nursing staff who did so, in all the six domains. Fewer nursing managers rated themselves less than 8/10, suggesting that they perceive themselves to be competent in management. In contrast, the majority of subordinates rated their managerial competencies less than 8/10. The financial management domain had higher percentages of nursing managers, supervisors, and subordinate nurses indicating scores less than 8/10, suggesting additional training is needed in this area. Scores of less than 8/10 were recorded from 57.3% of subordinate nurses (*n*=59) and 54.1% of supervisors (*n*=53) for nursing managers in the problem-solving domain.

**Table 4:** Proportion of nursing managers rated below 8/10

Dimension	Nursing manager (*n*=105)	Supervisors (*n*=98)	Other nurses (*n*=103)
Communication	28 (26.7%)	58 (59.2%)	55 (53.4%)
Leadership and management	31 (29.5%)	52 (53.1%)	51 (49.5%)
Staff management	28 (26.7%)	58 (59.2%)	58 (56.3%)
Financial management	51/102 (50.0%)	71 (72.4%)	60 (58.2%)
Planning and priority setting	32/103 (31.1%)	61 (62.2%)	53 (51.5%)
Problem-solving	30 (28.6%)	53 (54.1%)	59 (57.3%)

## Discussion

We conducted a cross-sectional study to evaluate the competencies of PHC clinic nursing managers in two South African provinces by using a 360-degree approach that included their supervisors and subordinate nurses. This discussion brings together and interprets the main findings from each of the six domains of the competency evaluation; it also outlines the recommendations that flow from these findings and concludes the research study.

### Financial management

Our results show that PHC nursing managers, supervisors, and subordinate nurses provided the lowest ratings for financial management competencies compared to other categories. Hence, there was agreement on the need for training, albeit at different levels. The need for additional training in financial management among nursing managers is not unique to South Africa (15, 42). In the UK and Australia, hospital nursing managers indicated that they lack confidence in financial management (18, 43, 44). Nursing managers working in various community settings in South Africa also struggle with financial management and lack autonomy where budgets are concerned (15, 18). A key recommendation contained in South Africa's NHI White Paper is decentralisation of budgets and finances to lower levels of the health system where nursing managers will play an important role in its implementation (35). Financial management training is needed for these managers, but as a minimum, national and provincial health authorities should involve them in the preparation of annual clinic budgets. PHC nursing managers can contribute to budgeting process as they are aware of service delivery and community needs.

### Communication

Nursing managers considered themselves to have high skills in communication contrary to their supervisors and subordinate nurses who indicated that they needed additional training. In other high-income countries, nursing management competencies include communication as critical for health systems strengthening (2, 6, 19–23). Notwithstanding study setting differences, our results are similar to a UK study among nursing managers which indicated additional training in communication as a need (43).

### Leadership and management

The leadership and management functions that nursing managers encounter daily involve decision-making, delegating work, problem-solving, communication, and acting with moral integrity (1, 15, 36). That only one-third of nursing managers indicated the need for leadership and management training in this study contradicts the findings of the job satisfaction survey among the same managers which found that the majority wanted training in leadership and management (15). The differences could be due to different instruments used, social desirability bias, or different understandings among nursing managers, supervisors, and subordinate nurses about appropriate leadership and management competencies. Nonetheless, the implementation of PHC reforms in South Africa necessitates nursing managers with good stewardship and leadership skills (17, 45). PHC nursing managers need opportunities to refine their leadership skills through personal reflection, mentorship, and formal training programmes. Such leadership and management development opportunities could also influence their job satisfaction (15).

### Staff management

Several nursing managers in our study perceived themselves to be competent in the staff management, but more than half of their supervisors and subordinate nurses indicated that these nursing managers need further development. Nursing managers’ self-ratings on the domain of staff management contradicts the findings from the diary and job satisfaction studies where they expressed difficulties in managing unplanned staff absences and human resources shortages (15, 36). Therefore, these PHC managers need training in staff management to steer complex health system changes in facing staff shortages (46). In Taiwan, improved staff management facilitates teamwork, boosts morale, and reduces staff resignations or career changes (47).

### Planning and priority setting

Our results suggest that nursing managers require additional training in planning and priority setting. These results could be explained by three factors. Firstly, numerous times during the day, nursing managers are forced to abandon managerial duties to provide direct care to patients (15, 36). Secondly, the diary study among a subset of these clinic managers found that their supervisors were unsupportive and often derailed the activities of these nursing managers through unannounced clinic visits and setting unrealistic targets and deadlines (36). This could explain the lower scores of the supervisors. Thirdly, the lower scores from subordinate nurses could be due to the fact that they perceive PHC nursing managers to have weak planning and priority setting skills as they bear the brunt of heavy workloads or other health system deficiencies (15, 36).

Our findings are supported by other studies. International studies examining PHC and hospital management teams found competency gaps in planning and priority setting, which have negative consequences for health service delivery (7, 25, 43, 48). Studies in LMICs note that planning and priority setting is a skill confined to lists of activities rather than coordinated processes with measurable outcomes and procedures for monitoring performance of PHC clinics (7, 49). Planning and priority setting skills are necessary for health systems strengthening to address deficiencies, but they have received insufficient attention in nurse education and training (50).

### Problem-solving

In contrast to the perceptions of nursing managers, their supervisors and subordinate nurses indicated the need for training in problem-solving. The differences in ratings on problem-solving among the three groups could be due to the fact that the delegation of authority to solve problems resides with the district health managers (15, 36). These PHC nursing managers had indicated in another study that they had problems getting basic operational requirements due to complex procurement systems that they could not override (15, 36). Another South African study with PHC facility managers indicated that health system deficiencies hamper their attempts to solve service delivery problems, deal with public, and community health issues (42). Although carried out in a different context, a Turkish study on problem-solving abilities of hospital nursing managers highlighted several shortfalls (27, 51). Nursing managers in Nicaragua (52), Uganda (53), Liberia (44), and the Gambia (54) who have effective problem-solving skills contribute to health systems strengthening and improve quality of care (51). Hence, continuing professional development programmes should include problem-solving skills for PHC nursing managers.

This study found that the majority of nursing managers perceived themselves to be competent (very high skills and no training needed) in the five competency dimensions of communication, leadership and management, staff management, planning and priority setting, and problem-solving (Table 1) when compared to the ratings of their supervisors and subordinate nurses. The higher self-rating scores by the PHC nursing managers were also found in another study (26), where employees overestimated their skills, making it difficult to give meaningful feedback (55). Other 360-degree evaluations have also found that individuals tend to rate their own competencies higher compared to their peers, supervisors, or those reporting to them (31, 34, 40, 55–57). An Iranian study that compared the competency assessments of hospital practising nurses with that of their head nurses found similar differences, with head nurses rating the practising nurses much lower compared to the nurses themselves (58). However, it is difficult to make direct comparisons with this Iranian study because of differences in methodologies used and different levels of health facility (PHC clinic vs. hospital).

In our study, both supervisors and subordinate nurses rated PHC nursing managers lower in all domains, and there were poor levels of agreement between the self-ratings of nursing managers and the ratings of supervisors and subordinate nurses. The ratings of supervisors and subordinate nurses suggest that the PHC clinic managers need additional training on all domains. These differences could be because of systematic rater biases (59), such as personal perceptions and experiences, which influence the rating process. The use of a Likert scale may have contributed to these results as it usually allows respondents to choose an option that best aligns with their perception (60). Objective measures to determine the competencies of nursing managers and combining that with self-perceptions of training are needed in future research.

Nonetheless, the 360-degree evaluation approach was useful in examining the perceptions and agreement of PHC clinic nursing managers among multiple raters, that is, nursing managers themselves, supervisors, and subordinate nurses. The 360-degree evaluation also increased the nursing managers’ awareness of the competencies needed in a PHC clinic, and the perceptions of others of these competencies (59). The low scores in financial management by the PHC nursing managers themselves suggest the need for additional training in this area.

Our study response rate was excellent. The results suggest a useful tool to measure PHC nursing managers’ competencies with high levels of internal consistency. Our tool, combined with more objective measures of competencies, could be relevant for measuring nursing managers’ competencies in other parts of South Africa or other LMICs settings, or to serve as a baseline for monitoring progress in the two study provinces. An overall factor analysis suggested that five factors explain the variability in the items; therefore, we might not need six domains. However, it goes beyond the scope of this study to conduct a psychometric analysis. Hence, the tool would need to be validated as the South African context and health sector reforms influenced the development of the tool.

## Conclusion

South Africa's health sector reforms based on PHC require competent nursing managers to ensure successful implementation of these reforms. Although the socio-demographic characteristics of PHC nursing managers show that they have extensive experience in the health sector, our study findings suggest the need for additional training in financial management. These skills in financial management could be enhanced through continuing professional development programmes. Furthermore, additional training must be aligned with health system goals and supported by a positive practice environment.

## Paper context

Previous research on this topic has focused on the competencies of district and hospital managers. This paper adds new knowledge regarding the managerial competencies of PHC clinic nursing managers in South Africa through the use of a 360-degree evaluation method. We recommend that the financial management competencies for PHC clinic nursing managers should be prioritised in continuing professional development programmes in South Africa. The tool developed in this study could be used in other low- and middle-income countries and complemented with the use of more objective measures of competence.
